# Looking Back, Moving Forward in Pain Medicine

**DOI:** 10.7759/cureus.44716

**Published:** 2023-09-05

**Authors:** Antonella Paladini, Christopher Gharibo, Sonia Khalbous, Ammar Salti, Tolga Ergönenç, Alberto Pasqualucci, Giustino Varrassi

**Affiliations:** 1 Life, Health, and Environmental Sciences (MESVA), University of L’Aquila, L’Aquila, ITA; 2 Pain Management, New York University (NYU) Langone Health, New York City, USA; 3 Anesthesia, Rabta Hospital, Tunis, TUN; 4 Anesthesia and Pain Medicine, Cleveland Clinic Abu Dhabi, Abu Dhabi, ARE; 5 Anesthesia and Reanimation, Morphological Madrid Research Center, Madrid, ESP; 6 Anesthesia and Reanimation, Akyazi Hospital Pain and Palliative Care, Sakarya, TUR; 7 Anesthesia and Critical Care, University of Perugia, Perugia, ITA; 8 Pain Medicine, Paolo Procacci Foundation, Rome, ITA

**Keywords:** opioid use, ultrasound guidance, interventional pain medicine, cannabinoids, pain, history of pain medicine

## Abstract

Pain is an ancient medical complaint and a clinical riddle that has never been entirely solved. Looking back into history was the springboard to a look into the future of pain medicine. This article was based on a series of presentations given in a recent congress (May 2023) and represents the research, views, and opinions of the authors.

Opium has been used for millennia to treat pain, but when it gained broad use in the United States in the 1980s and 1990s, it was so vastly overprescribed and mis-prescribed that it led to a public health crisis. This, in turn, led to the reaction where opioids at times were under-prescribed, leaving out many patients who may have benefited from opioids while leaving many legacy pain patients to manage withdrawal on their own and with few analgesic options. Cannabinoids (CB) were likewise widely used for various conditions, including pain, but were outlawed in the 20th century, only to be brought back as a potential analgesic agent. Interventional pain medicine is a developing discipline and has reinforced the concept of the interdisciplinary pain clinic. It plays an increasingly important part in modern medicine overall, especially with the support of ultrasound, for both diagnosis and therapy. Today, the views about pain have changed. Anyone has accepted that pain is not purely a physical phenomenon but a biopsychosocial phenomenon that occurs within a cultural context.

Pain management remains a small but vitally important medical subspecialty that is critical from a functional enablement and population health perspective, which is helping to navigate new therapeutic targets, new drugs and routes of administration, greater understanding of pain psychology, and new technologies.

Pain control today means early intervention, functional enablement through pain alleviation, educating patients about pain management, and minimizing the transition from acute to chronic pain.

## Introduction and background

The costs of pain are challenging to measure. First, there is the cost of human suffering, which is difficult to quantify. Then, there is the loss of human potential. In the United States, lost productivity due to pain amounted to over US$335 billion, eclipsing the direct healthcare costs attributed to pain at US$330 billion [1]. Pain was, is, and remains one of the most frequent clinical complaints, but the approaches to pain have undergone fundamental changes as the understanding of its mechanisms has expanded. And yet, the new understanding sometimes takes us back to ancient ways and methods.

Acute pain is often defined by its duration, often has an expected course, and resolves as the underlying injury or tissue heals. A setback occurs when acute pain transitions into the more challenging condition of chronic pain. Chronic pain, frequently defined by a duration of more than three to six months or pain persisting longer than it needs to be present, has fundamental mechanistic distinctions from acute pain [2]. The epidemiology of chronic pain is disputed, and depending on how it is defined, more than 20% of the US general population experiences chronic pain [3]. Also, 8% of the US general population experiences what has been termed “high-impact chronic pain” or conditions that impose severe limitations in the life domains of work, family and social life, and recreational activities [3]. Even worse results have been reported in many other studies [4], but it may impose a greater burden on low-income and developing nations [5]. Pain has remained a near constant in medicine since the very beginning, and despite its prevalence, our understanding of pain and our knowledge of how to treat it continue to evolve, remain controversial, and likely will continue to change for the foreseeable future.

For millennia, physicians have used opium and marijuana to treat many conditions, including pain. As botanical substances, it is difficult to know the potency and dosing of these agents, but they are recorded, albeit imprecisely, in centuries-old medical texts [6]. After falling out of favor with the advent of pharmaceutical products in the early 20th century, these agents first became illegal, and then, even their possession was criminalized. The emergence of opioids and cannabis in the advent of 21st-century pain care speaks to the diligence of pain medicine to seek out optimal solutions for analgesic relief. Interventional pain care, such as nerve blocks and device-based neuromodulation, is a newer approach that continues to evolve and still remains incompletely elucidated. Pain remains one of medicine’s oldest unsolved riddles, but merging old with new offers some helpful insights.

## Review

Methods

This article is based on a series of academic presentations given in Tunis, Tunisia, on May 11, 2023. The article is based on presenters’ talks, slides, and references and their own clinical experiences and opinions.

Results

Advances in pain care have sometimes occurred as pain specialists are willing to look back into history. Older methods of alleviating pain still have much to teach us. Not everything that is old is out of date; not everything new is great.

Looking back

From Opioid Analgesia to Opioid Crisis

The United States has had a unique and devastating experience with opioid analgesics, and the lessons learned in the United States may be of value to other nations. It began with a zealous endorsement. In 1980, a prestigious medical journal published correspondence revealing that of 11,882 hospitalized patients treated with at least one opioid analgesic product, only four patients without a prior history of addiction developed opioid use disorder. In fact, the journal stated that for patients with no history of substance use disorder, the risk of opioid addiction was minimal [7].

“Opioid pseudo-addiction” was introduced as a diagnostic term in a clinical note based on a single case where an individual exhibited addiction-like behavior that resolved with opioid dose escalation. The authors further went on to describe the “natural history of pseudo-addiction,” essentially establishing a newly coined disease based on a single case report [8].

In 1990, a “perspective” piece declared that opioid education was not enough and that narcotic regulatory practices need to be liberated for further progress so that unrelieved pain can be addressed by prescribing more opioids [9]. These articles were published in prestigious journals, but none was based on any sort of clinical study: they were a letter to the editor, a clinical note based on a case report, and a perspective piece or editorial. Yet, they helped shape how positively and favorably the American healthcare system related to opioid pain relievers and how little attention was given to the lack of foundation and the prevalence of opioid use disorder. Opioids were greeted around the turn of the millennium with unfettered American optimism (Table 1) [10-12].

**Table 1:** Opioids were used at higher doses and for longer periods of time than necessary, resulting in a series of cascading adverse consequences. The table was originally made by the authors. MME: morphine milligram equivalent

Results of the overutilization of opioids	Consequences
Opioid-associated effects, such as constipation, vomiting, and dysphoria	Often, treatment-limiting side effects and decreased patient satisfaction
For hospitalized patients: altered mental status, somnolence, dizziness, delayed ambulation, impaired rehabilitation efforts, and deconditioning	Increased healthcare resource utilization, increased hospital length of stay, increased costs, excessive daily MME, excessive discharge prescription, and requests for inappropriate post-discharge refills at the office
Inpatient “failure to thrive”	Delayed discharge can increase costs and low satisfaction with treatment
Opioid tolerance	Acute tolerance can reduce the analgesic effect, both acute and chronic pain patients can experience hyperalgesia and myalgia, and dose escalation to preserve the analgesic effect
Patients taking high doses of opioids due to prolonged use, tolerance, and inappropriate prescribing choices	Risk of side effects, tapering and cessation may involve withdrawal symptoms, patients may have to taper, requiring close clinical supervision, and limited options for patients to find alternative pain relief

Despite the problems caused by the overuse of opioid analgesics, physicians looking to nonsteroidal anti-inflammatory drugs (NSAIDs) were warned about side effects, including potentially life-threatening cardiovascular adverse events even in those with no history of heart disease [13,14]. NSAIDs are often contraindicated in elderly patients, leaving geriatric pain patients with limited options [15,16].

In 2016, the Centers for Disease Control and Prevention (CDC) issued guidance for primary care physicians about opioid prescribing [17]. Since that time, the total opioid-related overdose deaths have increased, but mortality associated with prescribed opioids remains the same, even though the prescribed opioid doses in morphine milligram equivalent (MME) dropped sharply [18-21]. The opioid epidemic had not resolved; it had only grown more complex. Prescription opioids were no longer the focal point of abuse; instead, many people with opioid use disorder were finding illicit sources of street drugs, such as heroin and fentanyl and its variants [20].

The Drug Enforcement Administration (DEA) reports that today, in the “third wave” of the American opioid crisis, illicit fentanyl is the significant driver of opioid mortality in the United States [22]. Since fentanyl and its variants are often obtained through illicit sources, this has shifted the opioid crisis away from legitimately prescribed opioids to street drugs [22-24]. Ironically, with this shift to illicit fentanyl, the CDC also “warned” prescribers about the dangers of opioids, particularly at high doses and/or as first-line treatment [25]. This impacted a large number of so-called legacy patients who had been treated for prolonged periods with high doses of opioids. Many were forced to abruptly discontinue long-standing prescriptions for opioids, with or without a taper, causing many to resort to illicit opioids with severe adverse or fatal outcomes, with worst outcomes in patients being associated with the longest use of opioids and the most abrupt tapers [26]. In those years, American healthcare has been accused of abandoning legacy patients [25] while at the same time pursuing its most egregious prescribers in criminal courts [27].

The extremes of the American experience, overprescribing and underprescribing, lead to a range of undesirable and certainly disastrous consequences that now appear to be correcting.

Cannabis

The official history of cannabis as a potential treatment for chronic pain dates back nearly five thousand years to China, where it appears in the Emperor Shen Nung’s pharmacopoeia, but it was widely used before that in Central Asia [28]. Cannabis is mentioned in the foundational books of Ayurvedic medicine in India and was described later by Greek, Roman, and Arabic physicians [29]. Cannabis was routinely recommended for a wide range of conditions, including pain, arthritis, inflammatory conditions, depression, asthma, epilepsy, and other conditions [6,30]. The rise of modern medicine spelled the end of many natural and botanical remedies, among them marijuana, which assumed a new identity as a dangerous intoxicant rather than a medical agent.

In the mid-19th century, Irish physician and renaissance inventor William Brooke O’Shaughnessy traveled to India and observed how cannabis stopped pediatric convulsions [31,32]. Through his efforts, cannabis was recognized as an important therapeutic product. There soon appeared case studies and reports on its use. In many parts of the world, cannabis products were available over the counter at drug stores and pharmacies and was recommended for many painful symptoms. In the United States, which would later criminalize marijuana, cannabis was listed in the official pharmacopoeia from 1854 to 1941.

The decline of cannabis as a medical product began in the mid-20th century in the United States with the Boggs and Narcotic Control Acts in the 1950s, culminating with the Controlled Substances Act of 1970, which relegated marijuana to Schedule 1, the classification for the most dangerous drugs with highest potential for abuse and no accepted medical uses [33]. At this same time, pharmaceutical products for pain relief such as aspirin came to market, eclipsing the analgesic role of cannabis [33]. With new synthetic pharmaceutical alternatives available, cannabis was deleted from the US pharmacopoeia, criminal penalties were imposed for even the possession of marijuana, and ironically, the recreational and therapeutic uses of cannabis increased in the United States [33]. The criminalization of marijuana may have had its root in racial overtones as well, as cannabis was described as a “jazz drug” used by Blacks, an intoxicant used by Mexicans, and a recreational substance used by hippies [34].

Regulating botanical products poses challenges for regulatory agencies, such as the Food and Drug Administration (FDA) of the United States. Unlike manufactured synthetic products, there were many different strains of the plant, and cannabis quality and potency varied markedly. Research into the drug isolated key constituents of marijuana: cannabinol (CBN), cannabidiol (CBD), and tetrahydrocannabinol (THC); endogenous cannabinoid (CB) receptors were discovered as were endocannabinoids [35]. This led to a “rethinking” about cannabis and its role in pain care and other therapies.

Internationally, attitudes about cannabis and cannabinoids are varied. Cannabis is legal in Canada and has a mixed but favorable status in most of Europe and Australia. In the United States, cannabis is illegal at the federal level but, confusingly, is legal in many individual states [36]. Cannabis remains illegal in Russia, China, and much of Africa [37]. Some countries, such as France and Italy, allow cannabis to be used for medical but not recreational use [38]. Some patients forego prescribed opioids in favor of marijuana, when it is available to them [39]. Although cannabis laws are in flux, it should be noted that the legalization of cannabis does not necessarily destroy the illicit market for marijuana; in the United States, for example, marijuana dispensaries exist side by side with illegal dealers, who may undercut prices [40].

Interventional Pain Medicine

It is difficult to pinpoint the start of interventional pain medicine, because any clinical effort to intervene and interrupt the pain cycle could technically be called “interventional pain management.” In medicine, there are surgical interventions, minimally invasive interventions, device-based interventions, and even pharmaceutical interventions. Exercise can be a form of rehabilitative intervention; increasingly, psychological interventions such as biofeedback are used. The goals of interventional pain care are the same as the global goals of pain medicine: to alleviate pain, restore function, enhance quality of life, and allow the patient to have as normal a life as possible. In addition, interventional medicine seeks to reduce or eliminate the use of opioids in achieving these goals. The World Health Organization’s (WHO) now iconic pain ladder for treating cancer pain in adults recommends mostly opioid analgesics, but interventional treatments could appear as a fourth rung, a treatment to consider when all others have failed [41]. But relegating interventional procedures as a treatment of last resort after extensive opioid treatments is unhelpful.

The history of interventional pain medicine may be said to have begun in the late 19th century with nerve blocks. The fundamental concept was that neural pathways carrying pain signals could be interrupted. The first such neural block was performed using cocaine, which numbed the tongue [42]. By 1901, caudal epidural injections were offered for pain relief, and in 1903, an alcohol block relieved trigeminal pain [43].

The next significant advance in interventional technique arrived with a new goal: diagnosing the source of the pain using interventional approaches [44]. The ability to identify a pain source facilitated treatment and allowed clinicians to differentiate etiologies among painful conditions with very similar presentations, such as low back pain. Dr. John J. Bonica, a pioneer in pain medicine, launched modern interventional pain care when he opened a multidisciplinary pain clinic in 1960 and advocated for not just pain control but also a greater and deeper understanding of the etiology and mechanisms of pain [45,46]. His premier multidisciplinary pain center started at the University of Washington and evolved into a clinic that employed 20 healthcare professionals in 13 different disciplines, all of whom were dedicated to alleviating pain. The concept Bonica launched assigned each patient a “manager” who examined the patient and then coordinated consultations and interpreted results [46]. The idea of pain as a multifactorial condition was launched.

How We Once Viewed Pain

The first major paradigm shift in our understanding of pain perception occurred in 1644 when René Descartes published a description of phantom limb pain, leading to his conclusion that peripheral pain was experienced not in the periphery but in the brain [47]. He was not the first to observe phantom limb pain, but he was the first to publish his conclusion that this was genuine rather than imaginary pain and offered a mechanistic explanation. The church, which often weighed in on scientific matters, believed that pain was rooted in original sin, and suffering could ennoble a penitent soul. Thus, in the early scientific exploration of pain, pain was considered either a sensory phenomenon or a spiritual, that is, psychological, one. This Cartesian dualism led early pain theorists astray and even today plays a role in the scientific thinking that severs the body from the mind in the experience of pain [48].

In the 18th century, Albrecht von Haller studied nerve fibers and discovered that they were the only thing that could produce sensation [49]. Pierre Jean Georges Cabanis introduced an emotional component to pain and stated that pain was largely beneficial because it preserved the equilibrium of the muscle system [49]. Finally, Xavier Bichat clarified the distinctions between the sympathetic and parasympathetic nervous systems [50]. Despite advances and experience, including electrophysiology, the dualistic nature of pain always lurked in the background, relegating pain to either a physical or a mental experience but not yet both.

Modern clinical considerations

Opioids

The WHO cancer pain ladder described pain simplistically and solely in terms of pain intensity [41], but there are different considerations as well, such as pain etiologies, ease of isolating and access to the anatomical site, pain characteristics, and mechanisms. Pain can often be a multi-mechanistic phenomenon. Depending on the mechanisms involved, pain may be effectively addressed with paracetamol (acetaminophen), NSAIDs, alpha-2 agonists, calcium channel blockers, antidepressants, ketamine, opioids, and spinal and peripheral interventions. Treating pain with a mono-therapeutic approach may fail to relieve even simple pain. Multimodal pain therapy is a recognized therapeutic strategy, particularly since it can have opioid-sparing effects [51].

A new paradigm for pain care recognizes pain as a biopsychosocial phenomenon and states that opioids are to be prescribed only based on a well-founded biological indication, such as cancer or postoperative pain, and to be used at the lowest effective dose for the shortest period of time [52]. Nonopioid analgesics and, when appropriate, nonpharmacological treatments can be combined.

Opioid rotation or switching from one opioid analgesic to another has been recommended to enhance tolerability, reduce side effects, and/or improve effectiveness [53]. This suggests that not all opioids work equally well for all patients, and there are important distinctions among drugs in this class. In some cases, this may be due to genetic variations causing differences in opioid analgesic response [54-56]. At any rate, a personalized approach to pain therapy is recognized today as the optimal prescribing strategy. It should be noted that a subpopulation of patients, possibly from 10% to 30%, find opioids ineffective and/or experience treatment-limiting side effects, including opioid-induced constipation, altered mental status, and dysphoria [57,58].

The 2016 guidelines from the CDC resulted in the drastic reduction of opioid prescribing but stranded some pain patients without good analgesic solutions [17,59]. The latest guidance from the CDC for opioid use to manage noncancer pain now emphasizes individualized care for pain patients that relies on clinical judgment and differentiates the needs of new patients from legacy patients; these guidelines also make dosing allowances for legacy pain patients and advocate their continuing treatment [60].

While pain physicians must optimize the use of nonopioid analgesics and consider opioid-sparing multimodal treatments when appropriate, opioids remain a valuable part of the armamentarium in pain care. When considering opioids for a patient, it is important to trial them with an exit strategy in mind in the event that opioids are not the optimal choice for that patient and set reasonable patient expectations regarding the risks and benefits of opioid analgesia.

Cannabis

Both scientific and social attitudes about cannabis changed with recent and rapid advances in our understanding of the endocannabinoid system, cannabinoid receptors CB1 and CB2, the chemical constituents of cannabis plants, and the greater elucidation of pain symptoms and disease models [61]. Cannabis contains over 450 compounds, of which at least 70 are phyto-cannabinoids. The two main cannabinoid compounds in marijuana are tetrahydrocannabinol (THC) and cannabidiol (CBD). THC is a psychoactive substance with analgesic effects, while CBD is both analgesic and anti-inflammatory but lacks psychoactive effects [62].

The endocannabinoid system is more complex. It involves 2-arachidonoylglycerol (2-AG), anandamide (ANA), THC, and CBD, which act upon the two receptors, CB1 and CB2. During pain or stress, an endogenous ligand is generated, having affinity for and activity at the cannabinoid receptors. The endocannabinoid system (endogenous cannabinoids, cannabinoid receptors, and associated enzymes) appears to play important roles in neurotransmission and pain signal processing by regulating nociceptive signaling in the periphery, in the dorsal horn, and in the supraspinal regions of the brain [63].

Cannabis use today may involve the botanical products or plant extracts, such as CBD oil. A number of products are commercially available in addition to botanicals. See Table 2.

**Table 2:** Cannabis-based medicines. Note that these commercial products have varying amounts of THC, the psychoactive component, with respect to CBD [64]. CBD, cannabidiol; THC, tetrahydrocannabinol

Generic name	Trade Name	THC/CBD ratio	Formulation
Nabiximol	Sativex	2.7:2.5 mg (~equal)	Sublingual spray
Semi-synthetic	Dronabinol (Marinol)	High THC/lower CBD	Oral tablet
Semi-synthetic	Namisol	98% THC	Oral tablet
Nabilone	Cesamet and Canames	Synthetic THC	Oral tablet

Medical marijuana can also be smoked, but dosing control in such cases may be problematic. Most dried cannabis products have THC concentrations around 15%, which is higher than the usual doses studied in the clinical trials [65]. Using dried marijuana with 9% THC, a recommended initial starting dose is a single inhalation from one marijuana cigarette per day, which can be gradually increased to one inhalation up to four times day, amounting to about 400 mg or half of a joint [65]. Caution must be urged for patients not to exceed recommended doses.

Systematic reviews of cannabis for its safety and efficacy in the treatment of chronic pain have produced equivocal results [66-69]. These mixed opinions have impacted national and international guidelines. In 2018, the European Pain Federation (EFIC) issued a position paper acknowledging the “uncertainties and controversies” about the use of cannabis for chronic pain patients [70]. As such, the EFIC considers medical marijuana as a third-line therapy for chronic neuropathic pain; in other pain syndromes, medical marijuana should be regarded as an individual trial, only after all other treatments have failed, ideally as an adjunct and a part of a multidisciplinary pain control regimen [70]. In 2022, an editorial described the “Bermuda triangle” of low-quality evidence, conflicting recommendations, and a variety of different products and different indications, which complicated and confused the use of medical cannabis [71]. Thus, cannabis-based medicines and medical marijuana may be considered for chronic cancer pain and/or chronic non-neuropathic noncancer pain if all established treatments have proven ineffective, but in each case, cannabis should be considered as an individual therapeutic trial.

The Canadian Agency for Drugs and Technologies in Health (CADTH) issued its guidance on the role of cannabis in treating chronic pain and reported that cannabis may be beneficial in treating chronic neuropathic pain, but the study results for the use of cannabis in specific diseases, such as fibromyalgia and musculoskeletal pain, have not been conclusive [72]. The CADTH recommends cannabis as a fourth-line treatment and advocates for the use of pharmaceutical cannabis products before trialing botanical products. Guidelines from the Canadian Pain Society for treating chronic neuropathic pain were issued that recognize cannabinoids as third-line analgesics [73]. A systematic review from China about analgesics for neuropathic pain advocated for their use in multimodal therapy and as fourth-line treatment [74]. A simplified guideline document for prescribing cannabinoids in a primary care setting limited the role of cannabinoids to neuropathic pain, palliative care, chemotherapy-induced nausea and vomiting, and various types of spasticity [75].

In particular, the role of cannabis in treating fibromyalgia remains controversial. Some studies recommend it [75,76], while others do not [73]. The role of cannabis for the management of pain associated with osteoarthritis, rheumatoid arthritis, cephalgia, back pain, and multiple sclerosis is unclear [70,72,77]. Cannabis may play an important role in treating migraines [78], and nabilone, specifically, has been found effective in reducing pain in patients with long-standing, intractable, medication-overuse headache [79].

A review of cannabinoids for the treatment of various types of chronic noncancer pain examined 104 studies (about 10,000 patients) and concluded that the effectiveness of cannabinoids for noncancer pain treatment is limited [80]. While larger and more rigorously designed clinical trials are needed, cannabis is not likely to emerge as a frontline analgesic for chronic noncancer pain.

Nevertheless, cannabis remained a popular topic of research and public interest. The International Association for the Study of Pain (IASP) convened a task force for a position paper on the role of cannabis in pain management. The systematic review the task force published evaluated 36 randomized controlled trials (7,217 patients) including both acute and chronic pain patients [81]. Unfortunately, the studies showed a high risk of bias, evidence was low quality, and adverse events were higher in the cannabis groups than controls. As a result of these findings, IASP at this time does not recommend cannabis or cannabinoids for any type of analgesia [81].

The challenges in cannabis research are formidable. There are many different products and formulations; botanical products are challenging to test in a highly controlled setting, and most research is not well funded and thus is done on a small scale. As a result, scientists, researchers, and prescribers have only small-scale studies at risk of bias to inform them. Large, well-designed, randomized clinical trials are needed to help better understand the clinical pharmacology of these compounds, their risks and benefits in pain care, and a better elucidation of dose effects and drug administration.

Interventional Medicine: Beyond the Needle

The advent of the multidisciplinary pain clinic and the opioid abuse crisis in the United States launched a renaissance in interventional pain care, which seemed to overcome many of the barriers in more conventional pain care approaches [44]. Interventional techniques offered novel ways to properly diagnose pain symptoms while delivering the treatment at the same time; recommend effective interventions; avoid exposure to potentially side effect-prone, toxic, or addictive drugs; and reduce the risks of surgical interventions. There are drawbacks to interventional approaches to pain management: Interventional methods can require multiple encounters, using a lot of time and effort; upfront costs can be high; reimbursement may be problematic; and outcomes depend on the expert skills of the clinician and the competency of the multidisciplinary team.

The two main types of interventions are spinal and extra-spinal. Extra-spinal interventions include joint injections, trigger-point injections, diagnostic and/or therapeutic nerve blocks, and the intravenous (IV) infusion of lidocaine or ketamine for pain [82]. Spinal interventions could be subdivided into those with diagnostic versus therapeutic goals. The evidence supporting diagnostic interventions is fair to good (62%) compared to therapeutic evidence (52%) [82].

**Figure 1:**
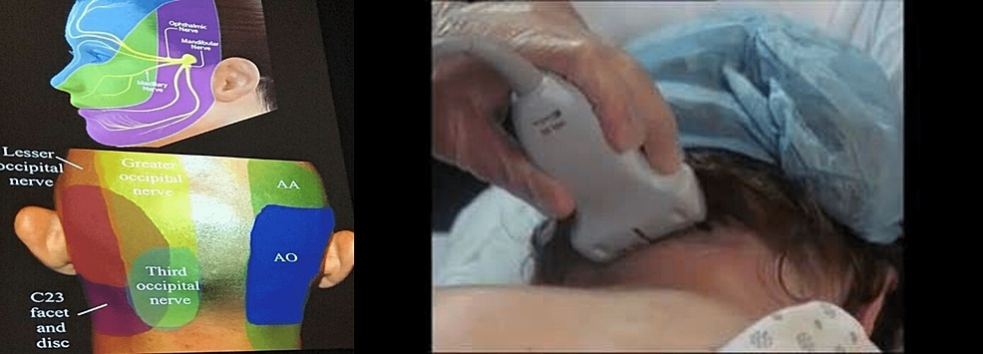
Treating pain at the third occipital nerve and C2-3 facet and disc using ultrasound guidance. In the top left image, the main nerves associated with face pain are indicated as they traverse the face. In the bottom left image, the nerves and C2-3 facet are shown from a posterior view. Note the lesser occipital nerve at the top and the third occipital never below. The right-hand image shows the ultrasound technician obtaining the images of these nerves and the facet and disc. AA, atlantoaxial; AO, atlanto-occipital

**Figure 2:**
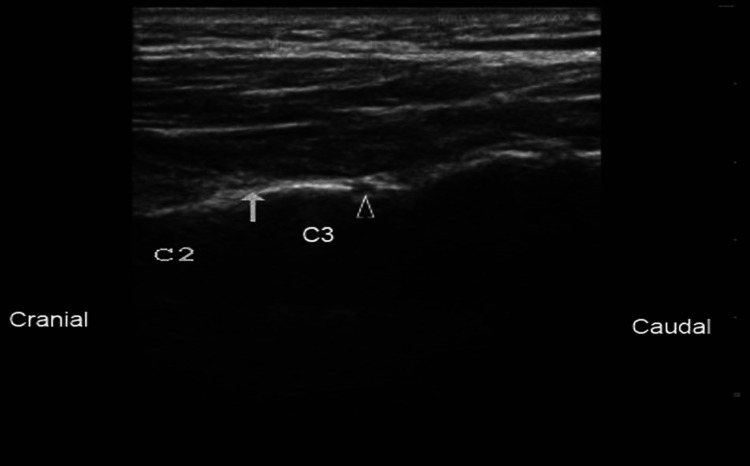
A view of the third occipital nerve obtained by ultrasound in the cervical spine from the same case as Figure 1. The arrows point to C2 and C3.

Ultrasound is readily available and inexpensive and can be readily deployed to help guide many interventional procedures. Figure 1 and Figure 2 are among the many examples of how advanced imaging has made greater and more precise interventions possible.

Interventional pain management can restore function, relieve pain, and improve the quality of life, but appropriate pain care starts with an accurate diagnosis. Only with an accurate diagnosis can a treatment be recommended and applied. It is important to remember that with all medical procedures, even the most familiar and least invasive, complications can occur. Complications require prompt recognition, treatment (which may involve a multidisciplinary team), appropriate documentation, and monitoring.

From the “Blind” Techniques to the Use of Ultrasound

Ultrasound has become an increasingly important tool in pain medicine in recent years. Traditionally, fluoroscopy has been the gold standard for guiding interventional pain procedures, but it has limitations, such as only visualizing bony structures. Conversely, ultrasound provides real-time, high-resolution images of soft tissues, allowing for more precise needle placement and reduced procedural complications. By combining ultrasound with fluoroscopy, clinicians can obtain a more complete picture of the patient’s anatomy and perform more accurate procedures.

The use of ultrasound in pain medicine has not been without its challenges. The early adopters of the technology faced a steep learning curve, as they had to learn how to interpret ultrasound images and integrate this information into their clinical practice. In the early days, nerve blocks were performed using the paresthesia technique, which involved inserting the needle until the patient felt tingling. This technique was associated with a risk of nerve damage [83]. Gentili and Wargnier [84] pointed out that several studies support the idea that paresthesia increases the risk of nerve trauma. As a result, they stated, “no paresthesia, no dysesthesia.”

In the 1970s, nerve stimulators were introduced as a more accurate method for nerve blocks. These devices use electricity to stimulate nerves so the practitioner can identify the needle’s correct location. However, ultrasound started to be used in regional anesthesia after the 1990s. The adoption of ultrasound in pain medicine required time for ultrasound technology to become more sophisticated and user-friendly.

As ultrasound technology advances, we can expect to see even more improvements in its use in pain medicine. New technologies, such as fusion imaging and artificial intelligence, are being developed to make ultrasound more accurate and effective. As these technologies mature, ultrasound will likely take its rightful place in pain medicine.

How We View Pain Today

Today, it is recognized that pain is not one thing, pain is a multidimensional experience, and there are many possible mechanisms that can cause painful symptoms. Most pain is mixed, that is, involves more than one mechanism. Treating pain must be preceded by a diagnostic identification of those mechanisms so that all of them can be addressed, whether if it is nociceptive, neuropathic, central, or psychological. Treating nociceptive pain with a neuropathic component must treat both types of pain mechanism, or the patient’s pain will be incompletely or perhaps not at all relieved [85]. Opioid monotherapy is discouraged today, because although it can be effective in the short and even long term, it needs to be done in a way that reduces harm and can still be associated with side effects, tolerance, and abuse potential. Equivalent analgesia with fewer adverse consequences may be obtained by using additive or synergistic combinations of smaller amounts of opioids and nonopioid analgesics, where nonopioids are reducing the dosages of opioids [86].

Although chronic pain is typically defined temporally, it actually differs mechanistically from acute pain. Acute pain is pain of shorter duration, but it is also a kind of pain that resolves as the underlying injury heals. Centralization can cause acute pain to transition to chronic pain, which is far more challenging to treat [87,88]. Preventing or mitigating that transition plays an increasingly important role in modern pain medicine. One way to prevent the transition from acute to chronic pain is to treat acute pain aggressively [87].

Discussion

Pain management is a small but vitally important medical subspecialty with an intriguing history. Many drugs being studied and evaluated for pain symptoms today have been known for millennia, yet their effects and side effects remain elusive to investigators. Opioids, for example, can be effective pain relievers but bring with them many risks and disadvantages including side effects, tolerance, abuse potential, and, in some individuals, limited effectiveness or worse hyperalgesia [89]. However, not all reconsidered old drugs are breakthroughs. Cannabis is historically important, but its current role in pain care is limited, and it should be viewed with caution [90]. Medical marijuana is definitely an important new agent in the armamentarium, but evidence supports its use only for relatively narrow indications, such as chemotherapy-induced peripheral neuropathy pain and epilepsy. Its role in other conditions, such as fibromyalgia and headache, is more controversial because evidence is mixed.

There is an important personalized aspect to pain care that is only starting to be investigated. The role of genetics, the microbiome, and other factors may be the next radical paradigm shift in pain medicine [91]. New therapeutic targets are being discovered [92]. There are many exciting new horizons in pain care, as well as revitalization efforts for the mainstays of pain care.

This review has several limitations. It is based on presentations delivered in May 11-13, 2023, in Tunis, Tunisia, as part of an international pain convention. These findings are not a systematic review of the literature but rather a narrative of several presentations given on the past, present, and future of pain medicine.

## Conclusions

Historically, the primary issue in pain care has been to alleviate pain; today, it has expanded to functional enablement, preventing the transition from acute to chronic pain, and relief of suffering. Old remedies for pain, such as opium and marijuana, have re-emerged and are being investigated, but all analgesics come with risks, as well as benefits. These “new old” drugs clearly have value, but the experience of the “opioid era” and evidence from modern clinical trials limit their use. The investigation of new drug targets for pain, a greater understanding of neuroplasticity and pain perception, genetic information, and personalized medicine, as well as technological advances, serve pain medicine patients today. Interventional approaches are becoming increasingly more refined and highly targeted and offer solutions to certain painful conditions that could not be effectively treated pharmacologically.
